# The esthetic surgical management of a submandibular fascial space infection of odontogenic origin

**DOI:** 10.1186/1471-2334-14-S7-P69

**Published:** 2014-10-15

**Authors:** Cristian Rotaru, Iulian Filipov, Lucian Chirilă, Mihai Săndulescu

**Affiliations:** 1 “Dan Theodorescu” Clinical Hospital of Oral and Maxillo-Facial Surgery, Bucharest, Romania; 2 Dental Concept Studio, Bucharest, Romania; 3 Carol Davila University of Medicine and Pharmacy, Bucharest, Romania

## Background

Odontogenic infections are common complications in dental practice and can sometimes spread through the cervical fascia and cause abscesses of the deep fascial spaces. Some of these abscesses can be life threatening. Early diagnosis of these deep fascial abscesses and aggressive antimicrobial and surgical treatment with extensive drainage are of utmost importance. The aim of this presentation is to show the clinical characteristics of a submandibular fascial space abscess and its esthetic, minimally-invasive surgical management.

## Case report

We present two cases of submandibular space abscesses that underwent surgical treatment using an esthetic incision with a through-and-through suction drainage of the pus collection, unidirectional lavage and intravenous antibiotics. This technique is best suited for young patients with esthetic requirements and with abscesses located in only one fascial space. However, using the esthetic approach, one must follow the same principles of the surgical treatment as for the classical incision: complete evacuation of pus, creating and maintaining a good postsurgical drainage. If these principles cannot be accomplished, classical incision must be considered.

## Conclusion

Because of the anatomical location of such abscesses in the facial and cervical areas, classical surgical management and debridement is, in some instances, unacceptable by the patient, and classical extensive drainage is not easy to achieve.

